# HemI: A Toolkit for Illustrating Heatmaps

**DOI:** 10.1371/journal.pone.0111988

**Published:** 2014-11-05

**Authors:** Wankun Deng, Yongbo Wang, Zexian Liu, Han Cheng, Yu Xue

**Affiliations:** Department of Biomedical Engineering, College of Life Science and Technology, Huazhong University of Science and Technology, Wuhan, Hubei, China; Beijing Institute of Genomics, Chinese Academy of Sciences, China

## Abstract

Recent high-throughput techniques have generated a flood of biological data in all aspects. The transformation and visualization of multi-dimensional and numerical gene or protein expression data in a single heatmap can provide a concise but comprehensive presentation of molecular dynamics under different conditions. In this work, we developed an easy-to-use tool named HemI (Heat map Illustrator), which can visualize either gene or protein expression data in heatmaps. Additionally, the heatmaps can be recolored, rescaled or rotated in a customized manner. In addition, HemI provides multiple clustering strategies for analyzing the data. Publication-quality figures can be exported directly. We propose that HemI can be a useful toolkit for conveniently visualizing and manipulating heatmaps. The stand-alone packages of HemI were implemented in Java and can be accessed at http://hemi.biocuckoo.org/down.php.

## Introduction

A good picture is worth a thousand words. Recent progress in high-throughput techniques, such as DNA microarray, next-generation sequencing (NGS) and quantitative proteomics, has increased the demand for the visualization of multi-dimensional and numeric data [1]–[3].

As an intuitive strategy, a heatmap can graphically visualize the matrix data by representing individual values with different colors. To estimate how many papers have heatmaps, we carefully curated all original research articles published in 2012 of five leading journals, including *Nature Biotechnology*, *Cancer Cell*, *Genome Research*, *Genome Biology*, and *Molecular & Cellular Proteomics*, and found that ∼30.4% (202 out of 664 papers) contain at least one figure for heatmaps (Table 1). We also manually checked the 202 papers, and observed that the methods for drawing heatmaps were not mentioned in up to ∼66% (134/202) of them (Table 2). For 68 remaining papers in which the tools were clearly stated, nearly ∼46% of them visualized heatmaps with the R language package (Table 2). However, considerable programming skills, which some researchers do not possess, are needed for using R. Also, we found the Java Treeview, an illustrator of microarray data [4], was used in ∼24% of the 68 papers. Although the Java Treeview doesn't perform any clustering analyses, a clustered data file in CDT format generated by other tools must be provided an input. To overcome this limitation, Seo *et al.* developed an interactive tool of Hierarchical Clustering Explorer (HCE) for both visualizing heatmaps and clustering the numeric data [5]. The heatmaps in HCE can be easily manipulated, whereas the artwork quality was yet to be improved. Moreover, although heatmaps can be accomplished by a number of commercial or non-commercial softwares such as GeneSpring GX, Mayday [6], Cytobank [7] and D3 (http://d3js.org/), these tools were not designed specifically for heatmap generating. Recently, an interactive heatmap viewer called jHeatmap was developed [8]. The tool is useful for the intuitive and interactive visualization of complex data in the form of heatmaps. However, no further manipulations, such as re-coloration and re-rotation, can be performed. Also, the visualized heatmaps cannot be exported for the publication proposes. Thus, the development of an easy-to-use toolkit for conveniently illustrating heatmaps and exporting publication-quality figures will be a great help for both bioinformaticians and experimentalists.

**Table 1:** Using frequency of heatmap.

Journal	Num. of papers	Num. of heatmaps^a^	Per.^b^
*Nature Biotechnology*	81	19	23.46%
*Cancer Cell*	106	40	37.74%
*Genome Research*	144	58	40.28%
*Genome Biology*	92	26	28.26%
*Molecular & Cellular Proteomics*	241	59	24.48%

To estimate *how* many papers contain heatmaps, we went through all original research papers (excluding reviews and other articles) published in 2012 of five leading journals as below.

a Num. of Heatmaps, the number of papers containing with at least one heatmap figure;

b Per., the percentiles.

**Table 2:** The summarization of the methods for illustrating heatmaps among the 202 papers published in 2012 on five leading journals.

Tools^a^	Num.^b^	Web link^c^
R	31	http://www.r-project.org/
Java Treeview	16	http://jtreeview.sourceforge.net/
MATLAB	7	http://www.mathworks.cn/products/matlab/
SPSS	4	http://www-01.ibm.com/software/analytics/spss/
GeneSpring	2	http://genespring-support.com/
MultiExperiment Viewer	2	http://www.tm4.org/mev.html
Cytobank	1	https://www.cytobank.org/
Heatmap Builder	1	http://ashleylab.stanford.edu/tools/tools-scripts.html
Integrative Genomics Viewer	1	http://www.broadinstitute.org/igv/
Matrix2png	1	http://www.chibi.ubc.ca/matrix2png/
Mayday	1	http://microarray-analysis.org
Processing	1	http://processing.org/
N/A^d^	134	N/A
Total	202	

a Tools, the name of used tools;

b Num., the number of papers that used the tool;

c Web link, the website of the tool;

d N/A, not mentioned in the corresponding papers.

## Method

In this work, we presented a novel software package of HemI (Heatmap Illustrator, version 1.0), which used a red, green, and blue tricolor in a 256 color mode. Given a selected color scale, the total color space will be automatically processed into a numerical matrix (768 rows * 3 columns) by Java. Then the inputted gene or protein expression data can be linearly normalized as below:
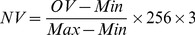
(1)More frequently, researchers prefer to visualize the logarithmic relations between different conditions and molecular expression levels. Thus, the original data can also be normalized as below:
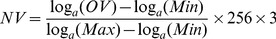
(2)While

- *NV* = normalized value
- *OV* = original value
- *Max* = the maximum of all OVs
- *Min* = the minimum of all OVs
- *a* = 2 (default), and can also be use-defined as 10 or e

In both equations, the *Max* cannot be equal to *Min*, and both *OV* and *Min* values must be greater than 0 in Eq. 2. The calculated *NV*s were then mapped to the color matrix, while the tricolor values of the nearest number of rows were visualized.

For further analysis of the data in heatmaps, several clustering approaches such as the hierarchical and *k*-means clustering algorithms, were also integrated. To calculate the distance, three types of linkage criteria (Table 3) and seven kinds of metrics (Table 4) were adopted for the two algorithms, respectively.

**Table 3:** Three mostly used linkage criteria for the hierarchical clustering.

Linkage criterion	Equation
Average linkage clustering (default)	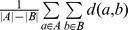
Minimum linkage clustering	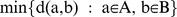
Maximum linkage clustering	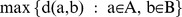

To calculate the pairwise distances for the hierarchical clustering, three commonly used linkage criteria were taken from the Wikipedia (http://en.wikipedia.org/wiki/Hierarchical_clustering).

**Table 4:** Seven distances for the clustering.

Distance	Equation
Euclidean distance	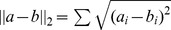
Squared Euclidean distance	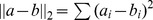
Manhattan distance	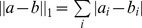
Maximum distance	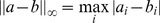
Pearson distance (default)	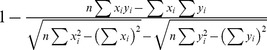
Spearman distance	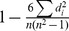
Kendall's tau distance	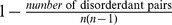

To calculate the distances for the hierarchical and k-means clustering approaches, up to 7 mostly used distances were adopted.

HemI 1.0 was written in Java 1.6 (J2SE 6.0) and packaged with Install4j 4.0.8. We developed six packages to support three major ×86/×64 operating systems (OSs), including Windows, Unix/Linux, and Mac. The stability and applicability of HemI was rigorously tested under Windows XP/7, Ubuntu, and Apple Mac OS X 10.5 (Leopard).

## Usage

Heml was developed in an easy-to-use mode. Here, we took data from a previously published study [9] as a demo to describe the usage of HemI. Androgen receptor (AR), a hormone-activated transcription factor, regulates prostate development, function and malignant transformation as an essential transcriptional repressor [9]. To characterize potentially AR-regulated genes, LNCaP prostate cancer cells were first hormone-starved for 3 days. Then, the gene expression levels were profiled after 3, 6, 12, 24 and 48 hours of androgen treatment [9]. Totally, Zhao *et al.* identified 428 androgen-repressed genes [9], and the corresponding data set was used as an example for HemI.

**Figure 1:**
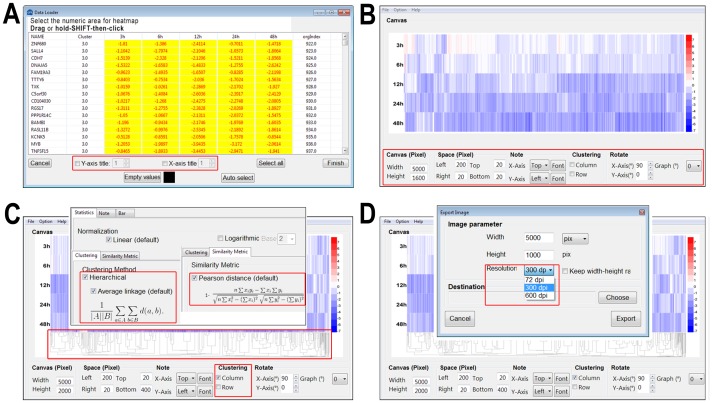
Usage of HemI 1.0. (A) The numerical data in one of three file formats can be directly loaded, whereas the data area can be selected by dragging or holding-SHIFT-then-click manipulations. Titles for *X*-axis or *Y*-axis can also be specified; (B) Multiple options for manipulating the heatmp; (C) The numeric data can be clustered for either or both of *X*-axis and *Y*-axis; (D) Publication-quality figures can be exported, and two figure formats were supported.

First, the numerical data in one of the three file formats, including Microsoft Excel spreadsheet (.xls), Tab Separated Value (TSV) or Comma Separated Value (CSV) can be loaded through clicking on the “LOAD” button of the main interface. Then, users can select the numerical data area for visualizing a heatmap with mouse-dragging or holding-SHIFT-then-click manipulations. The titles for *X*-axis and *Y*-axis can be specified by inputting number of row and column in the data sheet (Figure 1A). For convenience, an “Auto Fill” button was provided, while the first column and row were regarded as the titles of *Y*-axis and *X*-axis, respectively. A heatmap will be automatically generated after clicking on the finish button.

The generated heatmap can be easily manipulated in a customized manner. For example, the width and height of the artwork can be adjusted, whereas the blank space out of the heatmap can also be changed (Figure 1B). The picture can be re-colored, re-rotated, and the *X*-axis and *Y*-axis can be interchanged (Figure 1B). Moreover, the corresponding data can be clustered for either or both of *X*-axis and *Y*-axis by clicking on the clustering options of main panel (Figure 1C). After all configurations are finalized, the new heatmap can be updated and displayed by clicking on the “REFRESH” button.

To obtain publication-quality figures, users can export heatmaps by right-clicking on the canvas and choosing the export option. Users can select different resolutions for outputting figures, such as 72 dpi, 300 dpi and 600 dpi (Figure 1D). Two picture formats, including.png and.tiff, were also provided to satisfy the different requirements. The whole procedure was carefully implemented into a video with ∼4 minutes on our website (http://hemi.biocuckoo.org/faq.php).

## Discussion

**Figure 2:**
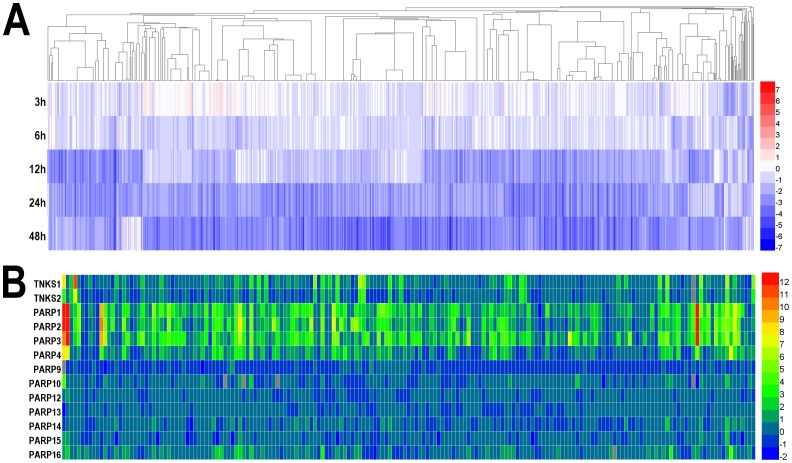
Illustrating heatmaps by HemI 1.0. (A) Thermal shifts, which indicate binding affinities of 185 compounds to 13 PARPs, were measured by DSF. A higher value represents a stronger binding affinity. (B) Totally, 428 androgen-repressed genes were identified from LNCaP cells, after the treatment of 1 nM synthetic androgen R1881 for 3, 6, 12, 24 and 48 hours. Values shown were normalized to 0 hour and log^2^ transformed.

The heatmap of potentially AR-regulated genes [9] was re-illustrated by HemI (Figure 2A). Also, poly-ADP-ribose polymerase (PARP) family proteins are involved in a variety of cellular pathways such as DNA repair and cell death, and regarded as a class of important drug targets in cancer therapeutics [10]. As a sub-family of PARP, tankyrases also play an essential role in telomere length regulation [10]. Recently, a differential scanning fluorimetry (DSF) approach was adopted for rapid profiling of 185 known and potential PARP chemical compounds for their binding ability to 13 PRAP family proteins including two tankyrases, TNSK1 and TNSK2 [10]. We redrew the heatmap of thermal shifts measured by DSF for all 185 inhibitors against 13 PARP members (Figure 2B). Our results are consistent with the previous analysis, which demonstrated that most of inhibitors lack specificity and mainly target PARP1-4 as primary hits, while several compounds can efficiently inhibit both PARP1-4 and two tankyrases [10].

Taken together, we propose that HemI 1.0 can be a useful tool for both experimentalists and bioinformaticians, and allow users to draw, manipulate and export publication-quality heatmaps in a user friendly manner. The software packages of HemI will be continuously maintained and improved upon users' comments and feedbacks.
